# Effective carbon partitioning driven by exotic phloem-specific regulatory elements fused to the *Arabidopsis thaliana AtSUC2 *sucrose-proton symporter gene

**DOI:** 10.1186/1471-2229-9-7

**Published:** 2009-01-20

**Authors:** Avinash C Srivastava, Savita Ganesan, Ihab O Ismail, Brian G Ayre

**Affiliations:** 1 University of North Texas, Department of Biological Sciences, PO Box 305220, Denton, TX 76203 5220, USA; 2 Samuel Roberts Noble Foundation, Plant Biology Division, Ardmore, OK 73401, USA; 3 Amyris Biotechnologies, Inc, Emeryville, CA 94608, USA

## Abstract

**Background:**

*AtSUC2 *(At1g22710) from *Arabidopsis thaliana *encodes a phloem-localized sucrose/proton symporter required for efficient photoassimilate transport from source tissues to sink tissues. *AtSUC2 *plays a key role in coordinating the demands of sink tissues with the output capacity of source leaves, and in maintaining phloem hydrostatic pressure during changes in plant-water balance. Expression and activity are regulated, both positively and negatively, by developmental (sink to source transition) and environmental cues, including light, diurnal changes, photoassimilate levels, turgor pressure, drought and osmotic stress, and hormones.

**Results:**

To assess the importance of this regulation to whole-plant growth and carbon partitioning, *AtSUC2 *cDNA was expressed from two exotic, phloem-specific promoters in a mutant background debilitated for AtSUC2 function. The first was a promoter element from Commelina Yellow Mottle Virus (CoYMV), and the second was the *rolC *promoter from *Agrobacterium rhizogenes*. *CoYMVp::AtSUC2 *cDNA restored growth and carbon partitioning to near wild-type levels, whereas plants harboring *rolCp::AtSUC2 *cDNA showed only partial complementation.

**Conclusion:**

Expressing *AtSUC2 *cDNA from exotic, phloem-specific promoters argues that strong, phloem-localized expression is sufficient for efficient transport. Expressing *AtSUC2 *from promoters that foster efficient phloem transport but are subject to regulatory cascades different from the endogenous sucrose/proton symporter genes has implications for biotechnology.

## Background

Phloem translocation is driven by hydrostatic pressure gradients between source and sink tissues. Many plants utilize phloem loading, the energized accumulation of photoassimilate in the collection phloem, to establish the high hydrostatic pressure, but pressure must also be maintained along the transport phloem for effective transport from source to sink tissues. Establishing and maintaining phloem pressure is therefore central to plant growth, and the concentrations of the major osmotically active solutes (sugars, amino acids, and potassium) are closely regulated (for recent reviews and discussion, see [1-4]).

In most plants, sucrose is the predominant osmolyte in the phloem and is accumulated by Suc/H^+ ^symporters [5]. Suc/H^+ ^symporters form a small gene family (*SUT*s or *SUC*s) in all species studied [6]. Group 2 family members, including *AtSUC2 *from Arabidopsis and Solanaceae *SUT1 *orthologs, are most prominently involved in phloem transport among dicots, whereas group 1 family members catalyze phloem loading in monocots [6]. These genes are developmentally and environmentally regulated to control the accumulation of sugar in the phloem. As examples, *AtSUC2 *induction in the minor veins follows the sink to source transition of developing leaves and requires light [7]; Solanaceae *SUT1 *genes are diurnally regulated [8,9]; and *BvSUT1 *is repressed in the phloem of *Beta vulgaris *(sugar beet) leaves by sucrose fed into the apoplast, indicating sucrose signaling [10]. At the level of post-transcriptional regulation, Suc/H+ symporters undergo rapid turnover [9] and are regulated by phosphorylation cascades [11], indicating that activity can be quickly altered. In addition, protein interactions between *Zea mays *(maize) SUT1 and proteins encoded by *TIE-DYED1 *and *TIE-DYED2 *are hypothesized to promote/regulate sucrose transport under high light intensity [12,13].

A physiological trigger regulating sucrose accumulation appears to be phloem hydrostatic (turgor) pressure [14]. For example, bathing a test system in hypertonic solutions of sorbitol to draw water out of cells and reduce pressure enhances sucrose uptake and acidification of the bathing solution, suggesting that both sucrose symporter and ATPase activity are stimulated [14-16]. Turgor-regulated Suc/H+ symporter activity in the phloem is consistent with findings that drought stress sufficient to affect photosynthesis has relatively little effect on translocation, since osmotic adjustment maintains pressure and transport [17]. In Arabidopsis, microarray experiments show modest increases (2-fold) in *AtSUC2 *expression in response to drought, abscisic acid (a drought induced hormone), or turgor stimulation [18]. Also, more effective sucrose transport during drought is implicated as an effective drought tolerance mechanism in drought resistant cultivars of *Phaseolus vulgaris *(common bean) [19]. In addition to sucrose, other solutes may accumulate during osmotic adjustment. In response to salt stress, for example, two phloem-specific polyol transporters increase expression in *Plantago major*, and the sorbitol to sucrose ratio in phloem exudates increases [20].

The role of *AtSUC2 *and Solanaceae *SUT1 *orthologs has been examined through highly informative, but relatively crude, mutation [21], suppression [22,23] and overexpression [24] studies. More recently, *AtSUC2 *gene activity was spatially restricted to the collection phloem to isolate its role in phloem loading from its role in long-distance transport, with the conclusion that during long-distance transport, it functions in retrieval from lateral tissues and not efflux to lateral tissues [25]. The extent to which the "fine-tuning" of symporter gene expression in response to environmental conditions contributes to plant growth and photoassimilate partitioning is not, however, addressed. The objective of this study was to drive *AtSUC2 *expression from different phloem-specific promoters in an *Atsuc2 *mutant background to establish whether exotic promoters can substitute for genomic sequences, because exotic phloem-specific promoters are unlikely to share identical patterns of expression. Commelina Yellow Mottle Virus (CoYMV) infects the monocot *Commelina diffusa *but contains a DNA element that confers strong, companion cell-specific expression to diverse species [26,27], and the *rolC *promoter from *Agrobacterium rhizogenes *is commonly cited as phloem specific [22,28]. Our results show that the strong *CoYMV *promoter adequately substitutes for the genomic *AtSUC2 *promoter, but that the weaker *rolC *promoter results in stunted growth and starch accumulation in the lamina of mature leaves. The potential for enhancing phloem transport or osmotic adjustment by expressing sugar-proton symporters from foreign or synthetic promoters is discussed.

## Methods

### Plasmid Construction

Plasmid constructions were by standard procedures [29] using *Escherichia coli *XL1-Blue (Stratagene, La Jolla, CA) as the host strain. Restriction endonucleases were from New England Biolabs (Beverly, MA), oligonucleotides were obtained from Invitrogen (Carlsbad, CA), and *PfuI *Ultra DNA polymerase (Stratagene) was used for PCR. All clones incorporating a PCR product were sequenced (SeqWright, Houston, TX). The starting material for new plasmids was pGEM::*CmGAS1p*::*cSUC2 *and pGEM::*SUC2p*::*cSUC2 *[25]. The Commelina Yellow Mottle Virus promoter (*CoYMVp*) from pCO1.Bam [26,27] was digested with *Pst*I and *Kpn*I and ligated into the same sites of pGEM::*CmGAS1p*::*cSUC2 *to create pGEM::*CoYMVp*::*cSUC2*. The *rolC *promoter from pBIN19::*rolC *[22] was digested with *EcoR*I, made blunt with Klenow, digested with *Kpn*I, and ligated with pGEM::*AtSUC2p*::*cSUC2 *digested with *Sal*I (made blunt) and *Kpn*I to create pGEM::*rolCp*::*cSUC2*. These promoter::*cSUC2 *cassettes where then subcloned into pGPTV-Bar [30], as is or as fusions with *GFP *and *uidA*, and electroporated into *Agrobacterium tumefaciens *strain GV3101mp90 as described [31].

### Plant Material

Wild type Col-0 and seeds of T-DNA insertional mutagenesis lines SALK_087046, SALK_001331 and SALK_038124 were obtained through the Arabidopsis Biological Resource Center [32]. Plant growth and genotyping were as described [25]. Heterozygous plants (At*SUC2*/At*suc2*::T-DNA; designated as At*SUC2 *+/-) were transformed by floral dip [33]. T1 generation seeds were sown on Sun Gro Metro-Mix 366 (Bellevue, WA) in 3.5 inch square pots (~1000 per pot), stratified for 72 hours, germinated in a controlled-environment chamber (Percival AR 95L, Percival Scientific, Perry, IA; 110–150 μmol photons m^2 ^s^-1^, 22°C/19°C, 14 h/10 h light/dark cycle), and transgenic seedlings were selected by spray application of glufosinate ammonium (20 mg L^-l^; "Finale", Farnam Companies, Phoenix, AZ) for seven consecutive days. Resistant plants were genotyped as wild type (*AtSUC2 *+/+), heterozygous (*AtSUC2 *+/-), or homozygous mutant (*AtSUC2 *-/-) by PCR with the RED Extract-N-Amp plant PCR kit (Sigma-Aldrich) according to the manufacturer's instructions, and using previously described oligonucleotides and PCR conditions [25]. Twelve or more lines independently transformed with the *AtSUC2 *cDNA constructs and segregating *AtSUC2 *-/- at the genomic locus were obtained from either the T1 or T2 generation.

For growth analysis, seed from Col-0, *AtSUC2 *+/-, and *AtSUC2 *-/- plants, and from the 12 independent lines for each construct (T2 or T3 generation) were germinated in individual cells of a 36-cell flat (T.O. Plastics, Minneaplois, MN). Plants were photographed 21 days post germination, just before the transition to flowering such that all aerial growth was represented in the rosettes. Rosette surface area (cm^2 ^plant^-1^) was measured with ImageJ version 1.38× [34]. For root measurements, representative lines were germinated on vertically oriented, square petri plates containing Murashige and Skoog medium with Gamborg vitamins (PhytoTechnology Laboratories, Shawnee Mission, KS), solidified with 5 g L^-1 ^gellan gum ("Gelrite", Sigma-Aldrich) and measured with a ruler 16 days post germination. XGlcA staining in plants transformed with *uidA *constructs was performed on 16 day-old whole plants using 3 mM potassium ferri- and ferrocyanide to limit diffusion of β-glucuronidase reaction products [31].

To measure transcript abundance, rosettes were harvested two week after germination to obtain two pools of approximately 50 mg for each line, except SALK_038124 *AtSUC2 *-/-, for which two pools of 5 mg were obtained. Isolation of total RNA, synthesis of cDNA with random hexamer primers, and semi-quantitative PCR, using oligonucleotides UBQ1 and UBQ2, and AtSUC2Ex3Ex4F and SUC2-3-ORF, to amplify *UBQ10 *and *SUC2 *transcripts, respectively, were as previously described [35].

### Carbohydrate Analysis and Radiolabeling

Major soluble sugars and starch were measured in the leaves and petioles of representative lines. Plants were grown for 30 days, and the first three adult leaves from siblings (n = 3 to 5) were excised at the stem and the fresh weight of lamina samples (leaf blade minus the midrib) and petioles measured; plants were processed between five and six hours after the beginning of the light period. Analysis of sugars and starch was previously described [25].

For [^14^C]-Suc and [^14^C]-Sorbitol uptake studies, intact rosettes of 14-day-old plants were harvested by cutting the hypocotyls, fresh weight was established, and plants were submersed in MES buffer (20 mM, pH 5.5 with KOH) plus 2 mM CaCl_2 _to prevent drying while other plants were processed. All material was harvested between six and eight hours of the illuminated period. Rosettes from each line were divided among three scintillation vials containing 5 mL fresh MES buffer with CaCl_2_, supplemented with either [^14^C]-Suc or [^14^C]-Sorbitol (1 mM; 30 KBq mL^-1^), and weighted down with 4 mm glass beads. Each replicate generally contained two or three pooled plants to obtain between 60 and 90 mg of intact rosettes, except SALK_038124 *AtSUC2 *-/-, which contained six to eight pooled plants to obtain 5 to 10 mg of rosette. The leaves were vacuum infiltrated for 5 min and incubated at room temperature with gentle agitation on a rotary shaker for 20 min, followed by three, 15-min washes in fresh buffer without labeled sugar. For each line, two of the infiltrated replicates were first cleared with 1 mL of 95% ethanol for 1 hour, and then bleached with 1 mL commercial bleach overnight. Five mL of scintillation fluid was added and [^14^C] uptake expressed as cpm (mg rosette fwt)^-1^. Plants in the third replicate were gently blotted dry after washing, placed between sheets of filter paper, and frozen in powdered dry ice. Frozen rosettes were lyophilized in a -30°C chamber for 48 h, pressed flat between steel plates in a large vice and exposed to X-ray film (Kodak BioMax MR Film, Rochester NY) for 36 h. Measurement of phloem exudation from radiolabeled leaves was as previously described [25].

## Results

### *AtSUC2 *mutants and growth habit of complemented lines

**Figure 1:**
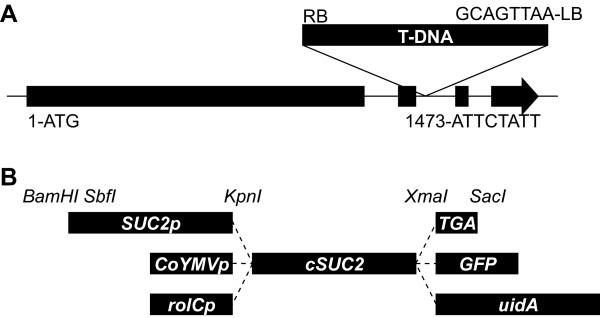
**Location of the T-DNA insertion in mutant line SALK_038124, and cassettes for expressing *AtSUC2 *from exotic promoters**. **A**, T-DNA insertion site in the second intron of *AtSUC2 *in SALK_038124 with sequences at the junction between T-DNA and genomic DNA indicated. Nucleotide numbering is relative to the ATG start codon, and based on gene model AT1G22710.1 at . **B**, Schematic representation of cassettes for expressing *AtSUC2 *cDNA (*cSUC2*) as a native protein and as fusions with reporter enzymes; promoters, fusions, and restriction endonuclease recognition sequences as indicated; TGA, in frame stop codon flanked by restriction sites for creating the native AtSUC2 protein. The figure is not to scale.

Three Arabidopsis mutants with T-DNA insertions at the *AtSUC2 *locus were previously described [21]. One of these (*Atsuc2-2*) contained a T-DNA insert in the first exon, and the other two (*Atsuc2-1 *and *Atsuc2-3*) each had an insert at different locations in the second intron. All three had the same phenotype: severe stunting, accumulation of starch and anthocyanin in leaves, delayed flowering, and failure to produce viable seed; all three were considered knockout mutations [21]. Two additional insertion mutants were recently characterized [25]. The insert in SALK_001331 is downstream of the *AtSUC2 *open reading frame and does not result in a visual phenotype. The insert in SALK_038124 is in a unique site of the second intron (Fig. 1A), and in the homozygous condition (*AtSUC2 -/-*), shows a phenotype similar to that described by Gottwald *et al*., (2000) [21]. SALK_038124 was further characterized for the presence of *AtSUC2 *transcript. Sequences from the third and fourth exons were not detected by semi-quantitative PCR, supporting the conclusion that SALK_038124 harbors a null mutation [25]. Based on this analysis, SALK_038124 is suitable for probing the function of AtSUC2 in whole-plant carbon partitioning by complementation with genes that have altered activity or expression pattern.

To test the ability of exotic promoters to drive *AtSUC2 *expression and distribute photoassimilate throughout the plant, *AtSUC2 *cDNA (referred to henceforth as *cSUC2 *to differentiate from the genomic locus, *AtSUC2*) was fused to the *rolC *promoter from *Agrobacterium rhizogenes *(*rolCp*) [22], the promoter element from Commelina Yellow Mottle Virus (*CoYMVp*) [26], and 2 kb of the *AtSUC2 *promoter sequence (*SUC2p*) as a positive control [25,36] (Fig. 1B). The stop codon of *cSUC2 *was flanked with *XmaI *and *SacI *restriction endonuclease recognition sites to create in-frame fusions with *GFP *or *uidA *(Fig. 1B).

Binary vectors carrying the *cSUC2 *cassettes were transformed into heterozygous *AtSUC2 +/- *plants because homozygous SALK_038124 (*AtSUC2 -/-*) plants are unsuitable for floral dip transformation. Transgenic progeny harboring these constructs were selected with glufosinate ammonium, and genotyped for segregation at the genomic locus as *AtSUC2 +/+, AtSUC2 +/-, or AtSUC2 -/-*. For each *cSUC2 *construct, 12 independently transformed lines were identified that were *AtSUC2 -/- *and, based on a 3:1 ratio of resistance:sensitivity to glufonisate ammonia in the subsequent generation, had the cDNA cassettes inserted at a single site. Tandem copies are possible.

**Figure 2:**
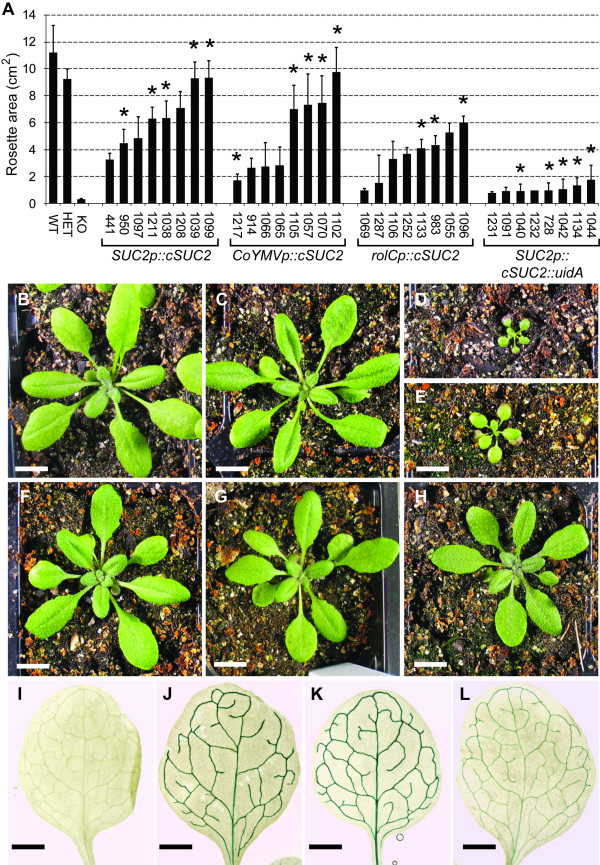
**Growth characteristics of controls and mutant plants complemented with promoter::*cSUC2 *cassettes**. **A**, Rosette area (cm^2^) of 21-day old wild type (*AtSUC2 +/+*), heterozygous (*AtSUC2 +/-*), homozygous mutant (*AtSUC2 -/-*), and homozygous mutant plants independently transformed (indicated by seed-stock number) with the indicated *cSUC2 *constructs; n = 4 to 10 sibling plants. Lines marked * are homozygous for the promoter::*cSUC2 *cassettes, and from these, a representative line for each was chosen for further analysis. **B to D**, representative 21-day old *AtSUC2 +/+*, *AtSUC2 +/-*, and *AtSUC2 -/- *plants, respectively. **E to H**, Representative 21-day old *AtSUC2 -/- *plants transformed with *SUC2p::cSUC2::uidA *(E; line 1042) *SUC2p::cSUC2 *(F; line 1039), *CoYMVp::cSUC2 *(G; line 1070), and *rolCp::cSUC2 *(H; line 1133) constructs. Scale bar, B through H = 1 cm. **I to L**, XGlcA staining in source leaves of untransformed wild type (I), and heterozygous *AtSUC2 +/- *plants transformed with *SUC2p::cSUC2::uidA *(J), *CoYMVp::cSUC2::uidA *(K), and *rolCp::cSUC2::uidA *(L). The staining pattern was the same irrespective of zygosity at the AtSUC2 locus. Scale bar, I through L = 1 mm.

The independent transformants demonstrated a range of growth, presumably reflecting differing levels of *cSUC2 *cDNA expression. Growth of eight independent lines for each construct is presented in Fig. 2A to show the range of complementation obtained. Those marked (*) were homozygous for the cDNA based on 100% resistance to glufosinate ammonia among seedlings (n > 16). The remainder had sensitive seedlings, showing they were still segregating for the cDNA in the generation used (T3 or T4), and the plants measured may have been hemizygous or homozygous for the transgene. Those harboring the promoter::*cSUC2::uidA *cassettes demonstrated poor growth, indicating that the β-glucuronidase fusion compromised AtSUC2 activity, but performed slightly better than the *AtSUC2 -/- *parent line (Fig. 2A, D, E), implying some activity *in planta*. β-Glucuronidase activity was not compromised, however, and these lines confirmed the expression patterns conferred by the promoters. Growth of plants with the promoter::*cSUC::GFP *cassettes was intermediate between *cSUC2 *plants and *cSUC2::uidA *plants (not shown), suggesting that AtSUC2 is somewhat tolerant of fusions proteins, but these GFP constructs were still not suited for complementation assays and were not pursued.

**Figure 3:**
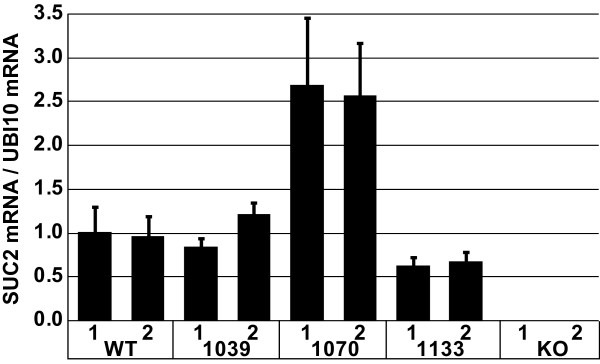
**Semi-quantitative RT-PCR of *AtSUC2 *and *cSUC2 *transcripts in wild type and experimental lines, relative to *UBQ10 *transcript (encoding ubiquitin)**. RNA was isolated from two pools of each line 14 days after germination and cDNA created by reverse transcription. Semi-quantitative PCR was performed in duplicate, and transcript levels expressed relative to *UBQ10 *transcript abundance. Variation is expressed as standard deviation among duplicates.

The average rosette area for the four most robust *SUC2p::cSUC2 *transformants was not significantly different from heterozygous plants (Fig. 2, Table 1) [25]. From these four robust lines that most closely mimicked wild type growth, a single line that was homozygous for the transgene was selected as a representative line for further study (Fig. 2F; line 1039). Two weeks after germination, *cSUC2 *transcript abundance in whole rosettes of line 1039 plants, relative to *UBQ10 *transcript as an internal standard [37], was very similar to that observed in *AtSUC2 *+/+ plants (Fig 3). Three weeks after germination, rosette growth in line 1039 was not significantly different from *AtSUC2 *+/+ or *AtSUC2 *+/- plants (Table 1). Root growth in line 1039 was not significantly different from wild type roots 16 days after germination on sterile MS media with 0% Suc (Table 1). A representative *SUC2p::cSUC2::uidA *leaf stained with XGlcA is shown in Fig. 2J, demonstrating staining only in the vascular tissue of mature leaves. In immature leaves, the staining pattern was characteristic of the sink-to-source transition, as previously described [7,36]. None of the 12 *SUC2p::cSUC2::uidA *lines analyzed deviated from this pattern (not shown).

**Table 1:** Measurement of rosette and root growth in control and complemented lines

	Rosette (cm^2^)		Root length (cm)
*AtSUC2 +/+*^a^	11.21 ± 1.98		5.23 ± 0.25
*AtSUC2 +/-*^a^	9.22 ± 0.76		n.d.^d^
*AtSUC2 -/-*^a^	0.28 ± 0.08^bc^		0.15 ± 0.10^b^

	Best 4 lines	Representative line	Representative line

*SUC2p::cSUC2*^a^	7.23 ± 1.41^b^	9.34 ± 1.26 (1039)	5.60 ± 0.70
*CoYMVp::cSUC2*	7.86 ± 1.26^b^	7.46 ± 2.00^b^ (1070)	4.17 ± 1.04
*RolCp::cSUC2*	4.91 ± 0.87^bc^	4.10 ± 0.61^bc^ (1133)	3.36 ± 0.44^b^

Measurement of rosette area (cm^2^) 21 days after germination among control and independently transformed lines grown on potting mix, and root length of representative lines grown for 16 days on sterile MS media without sucrose. Variation is standard deviation, n = 3 to 10 sibling plants.

^a ^[25]

^b ^Students T-test, p < 0.05, relative to wild type *AtSUC2 +/+*

^c ^Students T-test, p < 0.05, relative to heterozygous *AtSUC2 +/-*

^d ^n.d.; not determined

Rosette growth of the four most robust *AtSUC2 -/- *lines harboring *CoYMVp::cSUC2 *was similarly not significantly different from heterozygous plants (Fig. 2, Table 1). To be consistent in selecting representative lines, a single line homozygous for the *CoYMVp::cSUC2 *transgene was selected for further study from these four most robust lines (Fig. 2G; line 1070). Transcript abundance of *cSUC2 *was greater in line 1070 than *AtSUC2 *abundance in *AtSUC2 *+/+ plants two weeks after germination (Fig. 3). However, rosette growth was slightly reduced relative to *AtSUC2 *+/+ plants but was not significantly different from AtSUC2 +/- plants three weeks after germination (Table 1). Root growth was marginally less than wild type on sterile MS media with 0% sucrose (Table 1; p = 0.03). The *CoYMV *promoter is specific for companion cells (Matsuda et al., 2002) [26] and a representative XGlcA staining pattern is shown in Fig 2K. None of the 12 independent lines deviated from this pattern. Comparison of XGlcA staining in the *CoYMVp::cSUC2::uidA *lines, relative to *SUC2p::cSUC2::uidA*, qualitatively supports the semi-quantitative RT-PCR results showing that *CoYMVp *is stronger than *SUC2p*.

The rosettes of the four most robust *AtSUC2 -/- *lines complemented with *rolCp*::*cSUC2 *were smaller than heterozygous plants (Fig. 2, Table 1), and from these, a representative line was selected for further study (Fig. 2H; line 1133). Transcript abundance of *cSUC2 *in this line was reduced relative *AtSUC2 *transcript in AtSUC2 +/+ plants two weeks after germination (Fig. 3), and rosettes were significantly smaller than both *AtSUC2 *+/+ or *AtSUC2 *+/- rosettes three weeks after germination (Table 1). Root growth was also reduced (Table 1). Qualitative XGlcA staining in 12 independent lines harboring *rolCp::cSUC2::uidA *suggested that *rolCp *is less strong than the *SUC2p *and *CoYMVp*, but that the pattern is vein specific (Fig. 2L).

### Transient carbohydrate distribution in the leaf

Suc is the predominant transport sugar in Arabidopsis [38], and when activity of AtSUC2 (or the Solanaceae SUT1 ortholog) is reduced, soluble sugars and starch accumulate [21,22]. To assess the effect of the different promoters on carbon partitioning, the distribution of the major forms of transport and storage carbohydrate were analyzed in the lamina and petiole of representative lines. Wild type, 1039 (*SUC2p::cSUC2*), and 1070 (*CoYMVp::cSUC2*) plants did not show different levels of Glc, Fru, or Suc in the lamina or the petiole (Table 2), as expected from their similar growth. Starch similarly showed no difference in the petiole samples, but in the lamina, starch was slightly elevated in 1039 plants and reduced in 1070 plants. Soluble sugars and starch were elevated in both the lamina and petiole of the *rolCp::cSUC2 *line (1133).

**Table 2:** Analysis of transient carbohydrates in control and complemented lines

Representative line	Carbohydrate	Lamina	petiole
WT	Glu	2.28 ± 0.95	0.51 ± 0.02
(Col-0)^a^	Fru	0.80 ± 0.36	0.14 ± 0.03
	Suc	1.72 ± 0.76	0.35 ± 0.07
	Starch^b^	59.53 ± 4.96	8.28 ± 3.68
	Total	64.33 ± 4.42	9.28 ± 3.79

*SUC2p::cSUC2*	Glu	1.40 ± 0.42	0.53 ± 0.15
(line 1039)^a^	Fru	0.42 ± 0.12	0.12 ± 0.02
	Suc	1.40 ± 0.40	0.35 ± 0.07
	Starch	84.60 ± 18.20^c^	12.02 ± 3.64
	Total	87.83 ± 18.17^c^	13.02 ± 3.60

*CoYMVp::cSUC2*	Glu	1.11 ± 0.22	0.55 ± 0.08
(line 1070)	Fru	0.41 ± 0.13	0.14 ± 0.03
	Suc	1.90 ± 0.07	0.43 ± 0.04
	Starch	46.55 ± 4.50^d^	10.92 ± 2.55
	Total	49.96 ± 4.20^d^	12.04 ± 2.56

*RolCp::cSUC2*	Glu	15.41 ± 4.24^c^	0.67 ± 0.15
(line 1133)	Fru	3.44 ± 1.05^c^	0.17 ± 0.04
	Suc	11.50 ± 1.79^c^	0.52 ± 0.03^c^
	Starch	547.63 ± 117.90^c^	34.50 ± 6.80^c^
	Total	577.97 ± 113.92^c^	35.87 ± 6.98^c^

Sugar and starch in the lamina (excluding midrib) and petiole of the indicated plant lines after 30 days of growth. The first three mature leaves from each plant were pooled for extraction, and variation (standard deviation) calculated from n = 3 to 5 sibling plants. Sugars levels are expressed as nmoles per milligram fresh weight, and starch is expressed as glucose equivalents.

^a ^[25]

^b ^Expressed as glucose equivalents

^c ^Significant increase relative to wild type; Students T-test, p < 0.05

^d ^Significant decrease relative to wild type; Students T-test, p < 0.05

### Loading and transport of [^14^C]-Suc

**Figure 4:**
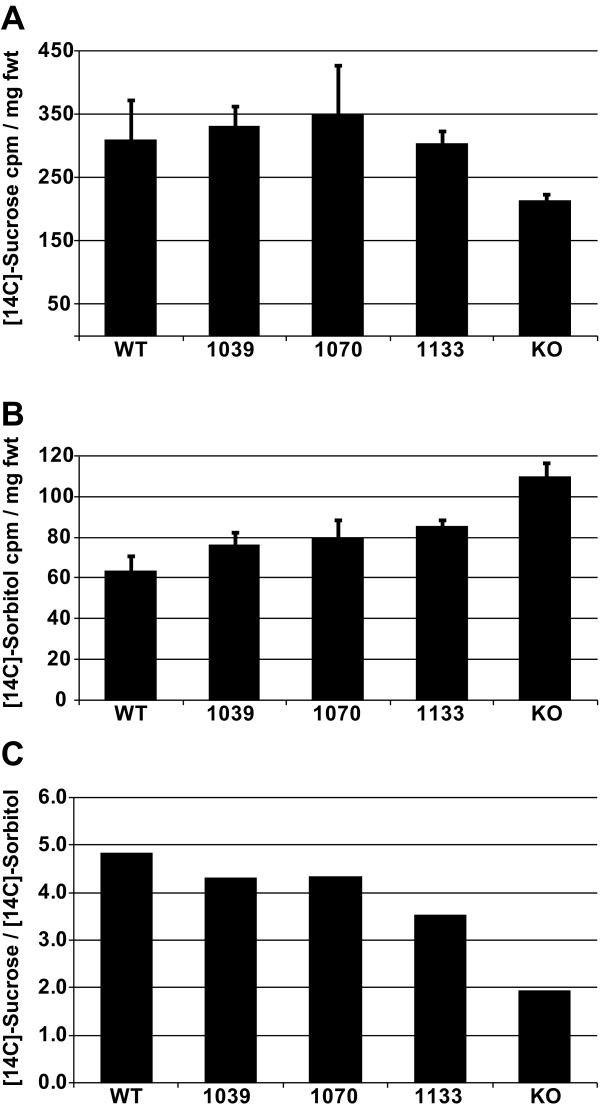
**Uptake of [^14^C]-Suc and [^14^C]-Sorbitol into whole rosettes of wild type and experimental lines**. **A**, Uptake of [14C]-Suc into whole rosettes, expressed as cpm per mg fresh weight; variation is standard deviation among duplicate samples. **B**, Uptake of [14C]-Sorbitol into whole rosettes, expressed as cpm per mg fresh weight; variation is standard deviation among duplicate samples. **C**, Uptake of [^14^C]-Suc in to whole rosettes, normalized against uptake of [^14^C]-Sorbitol.

**Figure 5:**
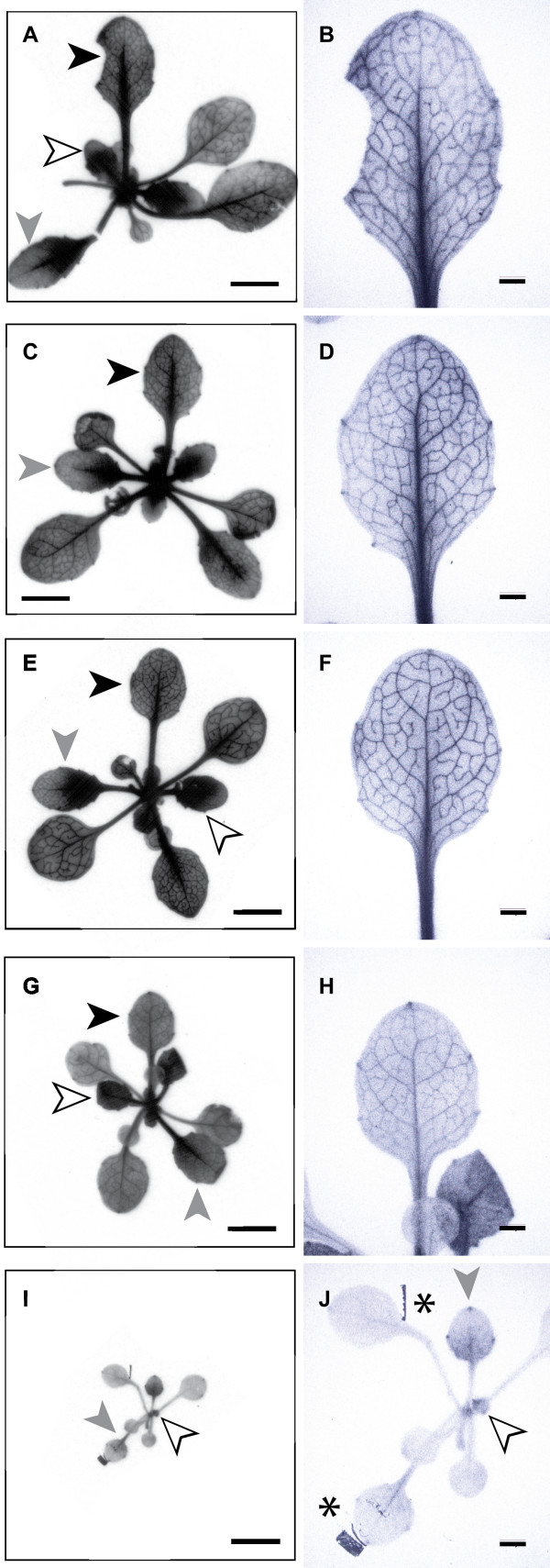
**Autoradiography showing accumulation of [^14^C]-Suc in whole rosettes**. **A through H**, Representative rosette and individual leaf from a wild type plant (**A, B**) and lines 1039 (**C, D**), 1070 (**E, F**), and 1133 (**G, H)**, respectively. Black arrowheads point to a mature source leaf with ^14^C accumulation in the veins and clearing from the areoles, white arrowheads point to sink leaves with diffuse labeling throughout the lamina, and grey arrowheads point to transition leaves. **I and J**, A representative homozygous SALK_038124 (*AtSUC2 -/-*) plant showing modest ^14^C accumulation only in putative sink regions of sink and transition leaves. Dark areas of the autoradiographs labeled (*) are artifacts of the adhesive used to secure the lyophilized tissue to a cardboard support in preparation for exposure to the X-ray film. The leaf directed to the top of the figure in A, C, E, G and I is magnified in B, D, F, H, and J; Scale bars, 5 mm in A, C, E, G and I 1 mm in B, D, F, H, and J.

To analyze Suc uptake in transgenic and control plants, rosettes were excised from the plant and infiltrated with a buffered solution of [^14^C]-Suc and incubated for 20 minutes. After thorough washing, two replicates were subjected to scintillation counting to quantify uptake (Fig. 4), and a third replicate was subjected to autoradiography to identify sites of [^14^C] accumulation (Fig. 5). Leaves from wild type controls (*AtSUC2 +/+*) and from the representative *AtSUC -/- *lines, 1039 (*SUC2p::cSUC2*), 1070 (*CoYMVp::cSUC2*), and 1133 (*rolC::cSUC2*) accumulated [^14^C] to similar levels, irrespective of differences in *SUC2 *transcript abundance, growth, and transient carbohydrate levels (Fig 4A). In addition, all showed label accumulation in the veins and clearing from interveinal tissues (areoles) of mature leaves (Fig. 5A–H). Label was distributed throughout the lamina of small sink leaves, and midsize leaves demonstrated vein labeling in distal portions and diffuse labeling in proximal regions of the leaves. The size and labeling pattern of these leaves strongly suggests that they are transition leaves, with distal regions loading and exporting sugar as source tissue, and proximal regions importing nutrients as sink tissue.

Homozygous SALK_038124 plants (*AtSUC2 -/-*), however, did not accumulate [^14^C]-Suc in a similar fashion (Fig. 4, 5). [^14^C] accumulation, relative to rosette fresh weight, was reduced in whole rosettes (Fig. 4A). In autoradiography images (Fig. 5I, J), enhanced signal was evident in sink leaves, and sink portions and hydathodes of transition leaves but not in the veins of mature source leaves. We considered that the small size of the AtSUC2 -/- plants may have compromised [^14^C]-Suc entry during vacuum infiltration or removal during washes, and a second experiment was conducted using [^14^C]-Sorbitol as an "inert" sugar (Fig 4B). If sugar entry and washing was independent of growth habit, then [^14^C]-Sorbitol retention per mg fresh weight was expected to be equal across all lines. [^14^C]-Sorbitol retention was however greater in AtSUC2 -/- plants (Fig 4C), suggesting non-specific retention in the AtSUC2 -/- plants is greater than the other lines. [^14^C]-Suc accumulation is expressed relative to [^14^C]-Sorbitol retention in Figure 4C, and emphasizes reduced Suc uptake in the AtSUC2 -/- line. Arabidopsis has at least one broad-specificity transporter that can use sorbitol as a substrate, but its physiological function is not clear and appears to be limited [39]. We cannot exclude the possibility of an increase in catalyzed sorbitol uptake in AtSUC2 -/- plants.

To assess the efficiency of photoassimilate transport out of the leaf via the phloem, excised leaves were photosynthetically labeled with ^14^CO_2_, and phloem sap was collected over 20 hours by an EDTA exudation method. At the end of the exudation experiment, the amount of isotope exuded from the leaves was determined relative to the amount retained by the leaves in soluble and insoluble fractions (Table 3). Leaves of wild type, 1039, and 1070 plants had similar distributions of label in the collected exudates and in both the soluble and insoluble fraction in the leaf. Leaves from *rolC::cSUC2 *plants (line 1133), however, exuded less label and retained more in the leaf soluble fraction (Table 3) consistent with this line having reduced levels of *cSUC2 *transcript (Fig. 3), reduced growth (Table 1), and elevated levels of soluble sugar in leaves (Table 2). All lines had equivalent levels of label in the insoluble fraction. This is not surprising since the exudation experiment was conducted over 20 hours in a humidity chamber under ambient room lighting to minimize EDTA entry into the leaf [25]. Under these low-light conditions, and over this period of time, any ^14^C initially incorporated into starch during the period of photosynthetic labeling (20 min) was likely converted back into soluble sugars.

**Table 3:** Exudation and retention of 14C in leaves after photosynthetic labeling

Plant Line	Percent Cumulative Exudation					Soluble Residual	Insoluble Residual
			
	0 hrs	2 hrs	4 hrs	9 hrs	20 hrs		
WT^a^	0.0 ± 0.0^b^	20.8 ± 4.1	36.7 ± 6.5	56.4 ± 6.2	70.1 ± 6.2	19.4 ± 4.9	10.5 ± 2.2
SUC2p (1039)^a^	0.1 ± 0.2	16.6 ± 5.3	31.4 ± 8.7	52.8 ± 6.5	66.5 ± 5.7	21.4 ± 1.8	12.1 ± 5.3
CoYMVp (1070)	0.5 ± 0.4^c^	14.3 ± 3.1^c^	27.8 ± 6.1^c^	51.4 ± 4.6	67.9 ± 4.2	19.9 ± 1.3	12.2 ± 3.0
RolCp (1133)	0.0 ± 0.0	15.1 ± 4.4^c^	26.8 ± 7.1^c^	47.0 ± 4.2^c^	62.6 ± 2.6^c^	24.4 ± 1.6^c^	12.9 ± 1.9

Percentage of ^14^C-photosynthate exuded from cut petioles and retained in the leaf 20 hours after photosynthetic labeling with ^14^CO_2_. Four mature leaves from each of four replicate plants were arranged in a 24 well microtiter plate and photosynthetically labeled for 20 minutes with 14CO2 in a sealed chamber three feet below a 400 W metal halide bulb. Exudation into 15 mM EDTA proceeded for 20 hours under ambient room light, and the EDTA solutions were changed and counted at the indicated time points.

^a ^[25]

^b ^Average ± Std. Dev., n = 4

^c ^Student's T-test, p ≤ 0.1 relative to WT.

## Discussion

Phloem loading is the thermodynamically unfavorable accumulation of solutes into the phloem [1]. The term refers to the accumulation of solute, principally sugars, into the minor vein phloem of mature leaves to accentuate the pressure gradients between source and sink tissues [40]. Phloem loading is a dynamic process that helps coordinate source capacity with sink demand. In plants that load Suc from the apoplast, expression of the necessary Suc/H+ symporter genes are regulated by development, light, diurnal cycles, sucrose signaling and response to turgor [7,9,10,13,41]. However the phloem also accumulates photoassimilate and other solutes during osmotic adjustment in response to changes in plant water status (e.g., [14-16]), and there is significant mechanistic overlap in the two processes. Early studies proposed that phloem turgor pressure was a principal regulator of both phloem loading for transport and solute accumulation for osmoregulation [42] and this is still an important component of more current models (e.g., see reviews [1,2,4]).

The objective of this study was to assess the ability of exotic phloem-specific promoters to substitute for the genomic *AtSUC2 *promoter in driving the expression of the *AtSUC2 *gene and promoting carbon distribution throughout the plant. Experiments were done in a line with a T-DNA insertion in the second intron of *AtSUC2 *[32] that had a phenotype consistent with three previously described T-DNA insertion mutants [21,25]. The T-DNA insertion greatly reduced transcript levels and deleted exons 3 and 4 from transcripts that were present [25]. We show in this work that [^14^C]-Suc does not accumulate in mature leaves (Fig. 5), further arguing that this is a null mutation. [^14^C]-Suc accumulation observed in sink tissues is likely mediated by one of the other family members [6]. Using this line, we recently showed that a promoter which confines expression to the companion cells of minor veins (*i.e.*, the collection phloem) was sufficient to restore Suc transport to *AtSUC2 -/- *plants [25]. In these experiments, the phloem-loading function of AtSUC2 was restored but functions in the transport phloem were not. Plant growth and carbohydrate distribution argued that AtSUC2 in the transport phloem is likely involved in Suc retrieval from the apoplast, and not efflux to the apoplast, but that even this retrieval function plays a relatively minor role in Arabidopsis growth and development [25].

The phloem-specific expression pattern for each promoter used in the current work has been described [7,26,36,40], but confirmation (Fig. 2I–L) is important for accurate interpretation of the results. Fusions between *AtSUC2 *cDNA and *uidA *or *GFP *did not restore satisfactory growth to mutant plants, but XGlcA staining in twelve independent lines for each promoter had the expected expression pattern (Fig. 2). Based on these results and previous characterization, there is no reason to suspect that any of the transgenes deviate from the expected expression patterns. Although *AtSUC2 *has been fused to reporter genes previously, and it was shown that fusions alter the cellular distribution of the protein [43], these studies were not carried out in a mutant background, and the activity of the fusion proteins was not gauged. This is the first effort demonstrating that fusions compromise symporter activity *in planta*.

Two kb of *AtSUC2 *upstream sequence was used as a positive control promoter. Two kb confers the same expression pattern as 3 kb, and gives strong phloem-specific expression consistent with the sink-to-source transition and the onset of phloem loading [7,36]. By expressing *cSUC2 *from this sequence in a mutant background, we functionally confirm that 2 kb is sufficient for effective phloem transport and robust growth. Line 1039 (*SUC2p::cSUC2 *control) had modestly elevated starch levels. Growth – although not significantly different from wild type – more closely resembled *AtSUC +/- *plants. Furthermore, *cSUC2 *transcript abundance was equivalent to that for *AtSUC2 *transcript in *AtSUC2 *+/+ plants. Characterizing more independent transgenic lines may resolve this discrepancy in growth, but it is also possible that the low levels of truncated transcript or protein (if produced) from the mutated gene may be interacting negatively with the transcript or protein from the cDNA transgene [25].

The experimental promoters, *rolCp *and *CoYMVp*, are similar to *SUC2p *in that both are active throughout the phloem and follow the sink-to-source transition [22,26]. *CoYMVp *is characterized as strong and companion cell specific [26]. In this work, we show that *CoYMVp *in line 1070 confers stronger expression than *SUC2p*, and plants expressing *cSUC2 *from this promoter showed robust growth and carbon partitioning. However, it is noteworthy that despite stronger *SUC2 *gene expression, line 1070 did not demonstrate enhanced growth but rather a modest reduction, and alterations in transient carbohydrate distribution and Suc loading and transport were not significant or very slight under laboratory conditions. This supports previous findings that Suc/H+ symporter activity is regulated both transcriptionally and post-transcriptionally [10,11].

The expression pattern conferred by the *rolC *promoter is not as rigorously analyzed, but it has been used extensively for phloem specific expression (e.g., [22,43]). In our hands, *rolCp *was not as strong as either *SUC2p *or *CoYMVp*, as determined by semi-quantitative RT-PCR and qualitative XGlcA staining, and this was reflected in reduced growth and the accumulation of soluble sugars and starch in lamina and petioles. Surprisingly, *rolCp *is activated by exogenous Suc, such that as Suc levels increase in the leaf, symporter expression should also have increased to promote transport [28]. Our findings that *CoYMVp *provides higher expression levels than *rolCp *is supported by previous findings [44].

Both *rolCp *and *CoYMVp *are from plant pathogens, *Agrobacterium rhizogenes *and a badnavirus that infects the monocot *Commelina diffusa*, respectively, and the results reported here suggest that phloem specificity and high levels of expression are sufficient for AtSUC2 (and orthologs) to mediate Suc loading and transport under laboratory conditions. However, although both promoters have presumably evolved phloem expression to favor interaction with their plant hosts, it is unlikely that their expression patterns are subject to the same regulatory cascades as symporters involved in phloem loading. For example, the *rolC *promoter is specifically activated by exogenous sucrose in tobacco [28] but the *BvSUT1 *promoter is specifically repressed by exogenous sucrose in sugar beet [10]. We are unaware of reports on *CoYMVp *regulation by sugar signaling or environmental simuli. Furthermore, Solanaceous SUT1 protein was recently localized to xylem parenchyma cells, suggesting a role in transporting Suc to transient storage reserves [45], but similar localization of *rolCp *and *CoYMVp *expression is not reported, and previous sequence comparisons did not identify similar sequences between these and other phloem-specific promoters [46].

The regulatory cascades that govern solute accumulation in the phloem evolved to ensure plant survival in natural environments, and these may not be optimal in domesticated crops where yield of harvested organs is the primary concern. As examples, Suc/H+ symporters are subject to diurnal regulation in the Solanaceae [9], are repressed by high Suc levels in sugar beet [10,11], and require a co-factor in maize under conditions of high light and Suc accumulation [12,13]. Each of these may contribute to balancing sink-source relations or may have protective roles but may also prevent available carbon from reaching harvested sinks. Although not addressed directly in this study, exotic or engineered promoters that do not reduce expression under these conditions may help maintain high levels of phloem transport and consequently contribute to increased productivity. Furthermore, since Suc accumulation is important for phloem osmotic adjustment, increasing the expression of Suc/H+ symporters during drought may enhance stress avoidance.

## Conclusion

Solute accumulation in the phloem is a dynamic process that helps coordinate the needs of sink tissues with the output capacity of source tissues, and through osmotic adjustment, maintains phloem hydrostatic pressure during changes in plant water balance. Sucrose uptake and the activity of the predominant phloem Suc/H+ symporter is regulated by numerous physiological and environmental stimuli, including leaf development, light, diurnal cycles, sugar signaling, and turgor. Despite this, exotic phloem specific promoters driving expression of *AtSUC2 *cDNA are sufficient to restore sucrose partitioning in *AtSUC2 *mutant plants to various extents. Because the endogenous Suc/H+ expression pattern may be sub-optimal for maximal carbon partitioning in new environments, the use of exotic or synthetic promoters to manipulate symporter gene expression and phloem loading may increase yield or osmotic stress tolerance.

## Competing interests

The authors declare that they have no competing interests.

## Authors' contributions

SG and IOI identified and characterized the Arabidopsis mutant lines; ACS, IOI, and BGA constructed plasmids; ACS transformed Arabidopsis and characterized complemented lines for growth and transient carbohydrate content; ACS and BGA prepared the manuscript; BGA did the uptake and exudation of labeled sugars, transcript analysis, and designed and coordinated the study. All authors read and approved the manuscript.
